# Editorial: Allergic sensitization in infants

**DOI:** 10.3389/falgy.2023.1339678

**Published:** 2023-12-04

**Authors:** Kirsi M. Järvinen, Nitya Jain, Daniel Munblit, R. J. Joost van Neerven

**Affiliations:** ^1^ Pediatric Allergy and Immunology, University of Rochester School of Medicine, Rochester, NY, United States; ^2^ Department of Pediatrics Mass General Brigham, Harvard Medical School, Boston, MA, United States; ^3^ Division of Care in Long Term Conditions, Florence Nightingale Faculty of Nursing, Midwifery and Palliative Care, King’s College London, London, United Kingdom; ^4^ Department of Paediatrics and Paediatric Infectious Diseases, Institute of Child's Health, Sechenov First Moscow State Medical University, Moscow, Russia; ^5^ Cell Biology and Immunology, Wageningen University & Research, Wageningen, Netherlands

**Keywords:** infancy, food allergy, FPIES, microbiome & dysbiosis, LPS, lung

**Editorial on the Research Topic** Allergic sensitization in infants

Allergic sensitization typically starts in early life and is determined by a complex interaction of genetic and environmental factors. As infants gain initial exposure to allergens in infancy (and even before birth, in the womb) allergic sensitization can begin to occur, leading to the development of allergy. The aim of this Research Topic in Frontiers in Allergy, entitled *Allergic Sensitization in Infants*, was to provide an overview of the current knowledge on the topic. The Research Topic was successful in eliciting manuscripts relating to the key factors contributing to an increased susceptibility to allergic diseases in infancy, covering lipopolysaccharide (LPS) hyporesponsiveness, dysbiosis, and the delayed introduction of “highly allergenic” foods in infancy.

The Review by Léon focuses on the role of the effect of environmental LPS exposure levels as well as immune hyporesponsiveness to LPS as factors that are associated with T helper (Th2) cell development. The author discusses how the effects of LPS on conventional dendritic cells (cDCs), especially type 2 cDCs, can promote Th2 responses. During primary sensitization, respiratory epithelial cells are activated and induced to produce alarmins such as interleukin (IL)-33, IL-25, and thymic stromal lymphopoietin (TSLP). These induce innate lymphoid cell (ILC)2s to produce IL-13 in the respiratory mucosa.

The granulocyte-macrophage colony-stimulating factor (GM-CSF) produced by epithelial cells in combination with IL-13 increases the expression of the transcription factors interferon regulatory factor (IRF)4, Krüppel-like factor (KLF)4, and signal transducer and activator of transcription (STAT)6 in cDC2. This leads to a reduction in IL-12 production. Low IL-12 levels during the activation of naïve T cells drive their development—through the induction of T follicular helper (Tfh)2 cells—toward the Th2 type. Ultimately, these results are in support of B cells to switch to production of IgE. Interestingly, in infancy, a malfunctioning GM-CSF-monocytic DC (moDC) axis conditions for LPS hyporesponsiveness, hindering the induction of T-bet expression on cDC2s, further contributing to the decreased IL-12 production by cDC2 and Th2 development.

This insight is putting the epithelial cell back in the spotlight as an important player in the first steps of the induction of sensitization (1) but also implies an important role for early life innate immune responses to bacterial components such as LPS. The latter is, for example, also seen in the epidemiological studies investigating the impact of rural environments, which indicate that exposure to bacterial components and the composition of the airway microbiome can play a role in reducing the development of respiratory allergies (2).

Studies thus far have demonstrated airway dysbiosis with allergic asthma but have not assessed whether lung microbial community dysbiosis has a functional effect on allergen sensitization. Bloodworth et al. have gone beyond associations of lung microbial composition dysbiosis with allergic lung inflammation and have demonstrated that the lung microbiome composition of the offspring of allergic mothers confers neonate responsiveness to allergens and the development of allergic disease in mouse models, suggesting that an early life airway microbiota dysbiosis may have a significant function in the development of wheeze and allergic asthma in children. Bloodworth et al. reported that a dysbiotic airway microbiota (high in Proteobacteria and low in Bacteroidetes) increases the immune responsiveness of mouse pups to an allergen. This effect was dominant and the transfer of dysbiotic lung microbial communities from neonates of allergic mothers to neonates of non-allergic mothers was sufficient to confer responsiveness to an allergen in the recipient pups. Interestingly, they could reverse this dysbiosis through the dietary supplementation of pups with α-tocopherol (αT), suggesting that dietary components may not only influence intestinal short-chain fatty acid (SCFA) production by the microbiota that prevent allergic sensitization (3) but can also influence the composition of microbial communities in the respiratory tract that are associated with allergy risk.

A recent systematic review suggested that the early introduction of highly allergenic foods may reduce the risk of food allergy development, such as peanuts, eggs, and possibly cow's milk (4). Marget et al. assessed the factors influencing the timing of allergen introduction in the U.S., including updated peanut introduction guidelines. The authors utilized the Gastrointestinal Microbiome and Allergic Proctocolitis (GMAP), which is a prospective observational cohort in suburban Massachusetts enrolled over a period spanning the pre-2017 and post-2017 guidelines. This allowed a comparison of introduction practices before and after the publication of NIAID's 2017 peanut introduction guidelines. Post-2017 guideline infants were more likely to have been introduced to peanuts by 9 months of age than pre-2017 guideline infants, with only a positive trend seen for egg introduction. Interestingly, a first child was more likely to have been introduced to peanuts earlier than a non-first child. Children of black and Asian origin were significantly less likely to have been introduced to peanuts or eggs as early as white children.

Finally, Pier et al. explored the lag in the diagnosis of food protein-induced enterocolitis syndrome (FPIES), which is a non-IgE-mediated food allergy characterized by delayed repetitive vomiting, as well as referral patterns and healthcare utilization. Utilizing a retrospective chart review in Upstate New York medical centers, the median time to diagnosis was longer in FPIES than in IgE-mediated food allergy, likely relating to the lower recognition of this manifestation of food allergy, especially in acute care settings, as evidenced by the fact that none of the referrals were from the ED and that the most common reason for referral was concern of IgE-mediated allergy (51%) but not FPIES. There was a statistically significant difference in race/ethnicity, with a greater proportion of Caucasian patients in FPIES than in the IgE-mediated food allergy cohort. This study demonstrated a lag in the diagnosis of FPIES and a lack of recognition outside of the allergy community.

These last two studies illustrate the ongoing disparity in the dissemination of guidelines and education about food allergy among caretakers and care providers, which can relate to the increased rates of food allergy seen in these populations. The Research Topic also highlights potential future strategies to overcome infant TLR hyporesponsiveness, as demonstrated in farming lifestyle communities that are protected from allergic diseases, as well as by maternal dietary interventions.
